# Intravenous Diuresis in Severe Precapillary Pulmonary-Hypertension-Related Right Heart Failure: Effects on Renal Function and Blood Pressure

**DOI:** 10.3390/jcm12227149

**Published:** 2023-11-17

**Authors:** Lyana Labrada, Carlos Romero, Ahmed Sadek, Danielle Belardo, Yasmin Raza, Paul Forfia

**Affiliations:** 1 Division of Cardiology, Temple University Hospital, Philadelphia, PA 19140, USA; lyana.labrada@tuhs.temple.edu (L.L.); ahmed.sadek@tuhs.temple.edu (A.S.); 2 Precision Preventive Cardiology, Los Angeles, CA 91024, USA; 3 Division of Cardiology, Northwestern University Feinberg School of Medicine, Chicago, IL 60611, USA; yasmin.raza@nm.org

**Keywords:** pulmonary hypertension, diuresis, right heart failure

## Abstract

In patients with right heart failure (RHF) and pulmonary hypertension (PH), classical teaching often advises cautious diuresis in the setting of ‘preload dependence’ to avoid renal injury and hemodynamic compromise. However, while this physiology may hold true in some clinical settings, such as acute ischemia with right ventricular infarction, it cannot necessarily be extended to PH-related RHF. Rather, in patients with precapillary PH and decompensated RHF, diuresis aimed to decongest the right heart and systemic venous system may be directly beneficial. This study aimed to evaluate the effects of diuresis on renal function and blood pressure in patients with severe precapillary PH. A retrospective chart review was conducted on 62 patients with severe precapillary PH admitted for decompensated RHF. The hemodynamic phenotype of these patients was characterized by invasive hemodynamics and echocardiographic data. Laboratory and hemodynamic data were collected at both admission and discharge. After large-volume diuresis in this patient population, there was an improvement in both glomerular filtration rate and creatinine. While there was a decline in blood pressure after diuresis, this was not clinically significant, given the blood pressure remained in a normal range with improvement in renal function. In conclusion, this study demonstrated that despite concern for preload dependence, significant diuresis in patients with acute decompensated RHF from precapillary PH is not only safe but beneficial.

## 1. Introduction

Patients with right heart failure (RHF) and pulmonary hypertension (PH) have typically been considered to be ‘preload dependent’ [1]. This anecdote is problematic for many reasons, not the least of which is that PH is conceptualized as a single entity rather than hemodynamic subtypes combined with a patient’s specific clinical scenario. It is often advised that diuresis be avoided or undertaken with extreme caution in the setting of PH to avoid hemodynamic compromise and renal injury by way of decreased cardiac output (CO) [2] and systemic blood pressure. However, while the concept of preload dependence has been proven in the setting of right ventricular (RV) infarction [3,4], the same is not true for patients with RV dysfunction secondary to precapillary pulmonary hypertension.

On the contrary, in patients with significant precapillary PH who present with acute decompensated heart failure and volume overload, the etiology of RV dysfunction is increased RV afterload rather than impaired intrinsic RV contractility. In this setting, decongestion with diuresis may be immediately beneficial through the reduction in right heart filling pressures, RV dimension, and the degree of TR with ultimate hemodynamic improvement [5,6]. In reality, it is not uncommon for these patients to receive fluids for hypotension on presentation, ultimately having an opposite and detrimental effect on the increasing right-sided filling pressures and the degree of TR. Further, while recently published guidelines do generally recommend the use of diuretics in the setting of RHF and precapillary PH, there are no cited studies to support this recommendation, and there is no comment on the effects on hemodynamics and renal function [7]. Therefore, we aimed to examine the effects of significant intravenous diuresis on hemodynamics and renal function in patients with acute decompensated right heart failure secondary to severe precapillary PH.

## 2. Materials and Methods

### 2.1. Study Objectives

The study aimed to examine the safety and effects of significant diuresis on both renal function and hemodynamics in patients presenting with acute decompensated heart failure secondary to precapillary PH.

### 2.2. Study Design

We performed a retrospective study on an electronic chart review of 62 patients with severe PH-related decompensated RHF with signs and symptoms of volume overload. The patients were admitted to a specialized advanced heart failure and PH service and treated with intravenous diuresis tailored to the clinical needs of each patient. Prior to the start of our study, the Temple Institutional Review Board (IRB) evaluated and approved our protocol and methods. The Temple IRB determined that all the criteria for a waiver of Health Insurance Portability and Accountability (HIPAA) authorization were met.

### 2.3. Patient Selection

Consecutive patients on inpatient Advanced Heart Failure and Pulmonary Hypertension Service at Temple University Hospital were reviewed from 2013 to 2021. Weekly sign-out documents were reviewed by a single investigator and screened for search terms, including heart failure, volume overload, intravenous diuresis, and pulmonary hypertension. We included patients with severe precapillary PH. All patients meeting each of the following criteria were included in the analysis: (1) age 18 years or older, (2) PH categorized as World Health Organization (WHO) group 1 or 3 PH and defined as mean pulmonary arterial (PA) pressure ≥ 20 mm Hg on cardiac catheterization and pulmonary vascular resistance (PVR) greater than 4 Woods Units (WU), and (3) admission for acute decompensated heart failure and intravenous diuresis. Patients were excluded if they had PH that was predominantly related to left-sided heart disease as evidenced by (1) a left ventricular ejection fraction (EF) < 40%, (2) significant mitral or aortic valve disease, (3) a PVR less than 4 WU, or (4) if the pulmonary capillary wedge pressure (PCWP) was >15 mmHg on cardiac catheterization. Patients were also excluded if they were not diuresed during the inpatient admission or if their admission was non-heart-failure related (for example, trauma, infection, elective procedures, etc.).

### 2.4. Baseline Characteristics Data Collection

Data regarding baseline demographic characteristics, including age, gender, height, weight, and body mass index, were collected through chart review for each patient. Clinical data, including pertinent co-morbidities, functional class, past surgical history, PH-targeted medical therapy, other medication regimens, and social history, were collected. Echocardiograms closest in temporal proximity to admission were reviewed. The time between the echocardiograms and the admission dates ranged from 0 days to 4.3 months, with a median of 7.5 days. The following qualitative and quantitative data points were collected: date of the study, right atrial size, left atrial size, septal contour, RV outflow tract systolic notching, RV size, RV function, tricuspid annular planar systolic excursion (TAPSE), left ventricular EF, and all regurgitant or stenotic lesions of the tricuspid, pulmonic, mitral, and aortic valves. An echocardiographic assessment was performed by an independent and blinded observer who was a board-certified echocardiographer with extensive experience in echocardiographic quantitation of the right heart and pulmonary hypertension. The echocardiographer was provided with a visual scorecard with illustrations of mild, moderate, and severe septal flattening. The coding of the degree of septal flattening was made by comparing the reference illustration card to the reference echocardiographic image of the individual patient. Right heart catheterizations (RHC) closest in temporal proximity to admission were reviewed. The time between RHC and the admission date ranged from 0 days to 23.4 months, with a median of 20 days. The following data points were collected: date of the study, heart rate, systolic blood pressure, diastolic blood pressure, mean arterial pressure, and hemodynamic pressure measurements, including right atrial, RV systolic, RV diastolic, PA systolic, PA diastolic, mean PA, and PCWP. Additionally, CO, cardiac index (CI), PVR, and systemic vascular resistance (SVR) were examined for each RHC.

### 2.5. Outcomes and Measures of Clinical Response

Laboratory data were collected on the day of admission and the day of discharge, including sodium, glomerular filtration rate (GFR), and creatinine. The initial documented systolic, diastolic, and mean blood pressure values were collected from the admission day and from the discharge day. Jugular venous pressure (JVP) exam values (cm H_2_O) were collected from the initial history and physical note and from the clinical note on the day of discharge. While exams documented by attending physicians were prioritized, if these were not available, JVP exams documented by resident or fellow physicians were included, as they were obtained under the direct guidance of attending physicians within the Heart Failure and Pulmonary Hypertension departments. In order to quantify the amount of diuresis performed throughout the duration of the hospitalization, data were collected on net fluid balance, weight loss, and maximum daily diuretic dose. The diuretic dose was reported in milligrams of intravenous (IV) furosemide or furosemide equivalents for patients receiving either Bumetanide or Torsemide. Other data points collected included length of stay, 30 day readmission rates, and death during admission.

### 2.6. Statistical Analysis

Data were analyzed using IBM SPSS statistics V.22 software (SPSS Inc., Chicago, IL, USA). Descriptive data for continuous variables are presented as means ± SEM or as medians (25% and 75%) when appropriate. Categorical data was compared using Fisher’s exact test. Comparisons between groups for continuous variables were performed using unpaired two-sample *t*-tests or the Mann–Whitney test, as appropriate. Analysis of group effects with repeated exercise measures was performed by comparing mean slope coefficients from individual linear regressions. Pearson correlation coefficients were also used for categorical variables. A *p*-value of <0.05 was considered significant.

## 3. Results

### 3.1. Baseline Characteristics

Table 1 and Table 2 summarize the baseline characteristics of the study cohort and the baseline characteristics specific to PH etiology and management. Data from 62 patients meeting the inclusion criteria were reviewed. Patients’ ages ranged from 30 to 85 (mean 65 ± 14.4 years). The majority of patients were female (83.9%) and White (61.2%). The majority of patients had WHO Group 1 disease (90.3%), and six patients (9.7%) had WHO Group 3 disease (Table 2). PH medical regimens, specifically on the day of admission, were reported. Seventeen patients (27.4%) were on no PAH-specific medical therapy at the time of admission; 45 patients (72.5%) were on some form of PAH-specific medical therapy on admission, including phosphodiesterase type 5 (PDE5) inhibitors (*n* = 44, 71%), inhaled, oral, or parenteral Prostacyclins (*n* = 30, 48.4%), endothelin receptor antagonists (ERA) (*n* = 27, 43.5%), and soluble guanlylate cyclase (sGC) stimulators (*n* = 8, 12.8%). There were two patients (3.2%) who remained on no PH medical therapy at the time of discharge, and both of these patients were Group 3 PH. Other pertinent medications taken concurrently included either an angiotensin-converting enzyme (ACE) inhibitor or an angiotensin-receptor blocker (ARB) (12.9%), mineralocorticoid receptor antagonists (27.4%), calcium channel blockers (6.5%), beta-blockers (33.9%), statins (41.9%), and immunosuppressants (8.1%). Of note, 12 patients (19.4%) had coronary artery disease, and 12 patients had a history of atrial arrhythmias (19.4%).

### 3.2. Echocardiographic and Hemodynamic Data

**Figure 1:**
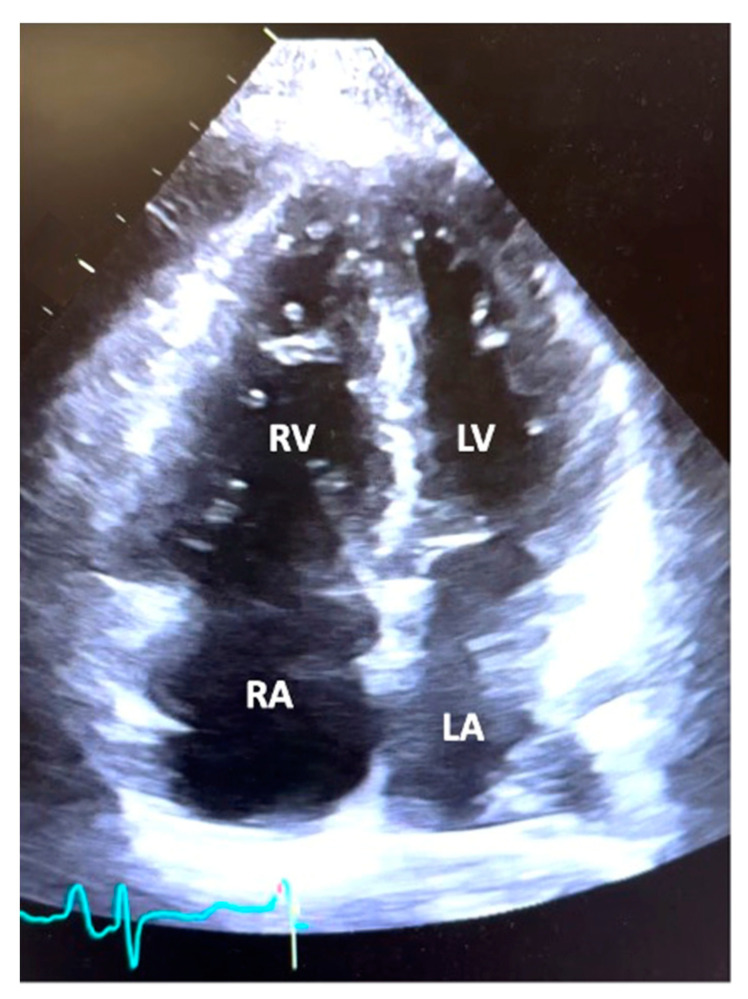
Echocardiogram. A representative image of an apical four-chamber view from a patient in the study demonstrating moderate right ventricular (RV) dilation, right atrial (RA) dilation, and normal left ventricular (LV) and left atrial (LA) size.

Table 3 and Table 4 summarize the echocardiographic and hemodynamic data from RHC, respectively. Echocardiography demonstrated normal left ventricular function, with a mean left ventricular EF of 60.9% ± 8.0 with a lack of left-sided valvular heart disease. The majority of patients had severe RV dilatation (66.1%) and severe RV systolic dysfunction (53.2%). The mean PA systolic pressure was 79.2 ± 22.2 mmHg, and the mean TAPSE was 1.4 ± 0.4 cm. RV outflow tract pulse wave Doppler systolic notching, a Doppler sign of elevated pulmonary vascular resistance and low pulmonary artery compliance, was present in 67.7%, absent in 19.4%, and not evaluated in 12.9% of patients. The degree of ventricular septal flattening was noted to be severe in 46.8%, moderate in 37.1%, mild in 9.7%, and absent in 6.4% of the patient population. Tricuspid regurgitation was noted to be either moderate or severe in 79.1% of subjects. Pericardial effusion was noted to be present in 46.8% of patients and mild to moderate in 90% of patients. Thus, on average, subjects had moderate to severe RV dysfunction, ventricular septal flattening, and tricuspid regurgitation. A representative image of the typical apical four-chamber view from a patient in the study is depicted in Figure 1.

The patients had severe PH by right heart catheterization, with a mean pulmonary artery pressure of 49.9 ± 10.9 mmHg and a mean PVR of 11.7 ± 5.7 WU. The mean PCWP was 10 ± 4 mmHg. Additionally, the mean right atrial pressure (RA) was 12 ± 6 mmHg, and the RA:PCWP ratio was elevated at 1.3 ± 0.7 [8]. Thus, most patients had a hemodynamic phenotype consistent with PH related to severe pulmonary vascular disease in the relative absence of left heart congestion [9,10]. Further hemodynamic data collected included RV systolic pressure (78.9 ± 16.5 mmHg), RV diastolic pressure (15.2 ± 8.1 mmHg), PA systolic pressure (80.0 ± 16.9 mmHg), PA diastolic pressure (32.8 ± 8.4 mmHg), CO (3.8 ± 1.2 L/min), CI (2.1 ± 0.6 L/min/m^2^), and SVR (1736 ± 824 Dynes-5).

**Table 4:** Right heart catheterization hemodynamics.

	Mean ± SD	Normal Values
Heart Rate (beats/min)	80.2 ± 14.0	60−100 beats/min
SBP (mmHg)	119.0 ± 24.2	90−140 mmHg
DBP (mmHg)	69.2 ± 13.7	60−90 mmHg
MAP (mmHg)	88.5 ± 17.3	70−105 mmHg
RA (mmHg)	11.8 ± 5.5	2−6 mmHg
RVSP (mmHg)	78.9 ± 16.5	15−25 mmHg
RVDP (mmHg)	15.2 ± 8.1	0−8 mmHg
PASP (mmHg)	70.0 ± 16.6	15−25 mmHg
PADP (mmHg)	32.8 ± 8.4	8−15 mmHg
Mean PA (mmHg)	49.9 ± 10.9	9−18 mmHg
PCWP (mmHg)	10.1 ±3.9	6−12 mmHg
RA: PCWP	1.3 ± 0.7	≤0.5
CO (L/min)	3.8 ± 1.2	4.0−8.0 L/min
CI (L/min/m^2^)	2.1 ± 0.6	2.5−4 L/min/m^2^
PVR (Woods Units)	11.7 ± 5.7	<2 WU
SVR (dynes/s/cm^−5^)	1736 ± 824	800−1200 dynes/s/cm^−5^

Abbreviations: CI—Cardiac index, CO—Cardiac output, DBP—Diastolic blood pressure, MAP—Mean arterial pressure, PA—Pulmonary artery, PASP—Pulmonary artery systolic pressure, PCWP—Pulmonary capillary wedge pressure, PVR—Pulmonary vascular resistance, RA—Right atrial, RV—Right ventricular, RVDP—Right ventricular diastolic pressure, RVOT—Right ventricular outflow tract, RVSP—Right ventricular systolic pressure, SBP—Systolic blood pressure, SVR—Systemic vascular resistance, TAPSE—Tricuspid annular planar systolic excursion, WU—Woods units. Normal Value References [NO_PRINTED_FORM] [7,8,10,11,12].

### 3.3. Admission Characteristics

Table 5 outlines admission characteristics. The mean admission duration was 10.1 ± 6.4 days, and the 30-day readmission rate was 12.9% (*n* = 8). Patients received relatively high dose diuretics, with a mean maximum daily diuretic dose of 552 ± 752 mg in oral Furosemide equivalents. Diuretic choices included Furosemide (25 patients), Bumetanide (41 patients), and Torsemide (2 patients). Patients experienced a mean net diuresis of 13.3 ± 7.6 L, with a mean weight loss of 7.0 ± 6.7 kg. Five patients (8%) received an additional diuretic, either metolazone or intravenous chlorothiazide. Fourteen patients (22.5%) had a new diagnosis of PH on admission. These patients were either directly admitted from a clinic after an initial outpatient visit or transferred from another institution in the context of a new diagnosis of PH.

### 3.4. Renal Function and Hemodynamics

**Figure 2:**
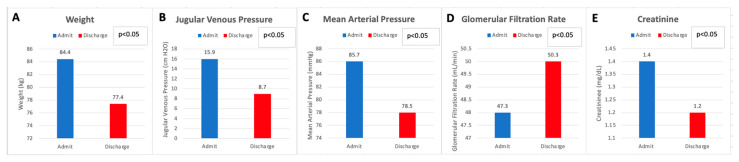
Pre- and Post-Diuresis Data. Admission and discharge values for weight (**A**), Jugular Venous Pressure (**B**), Mean Arterial Pressure (**C**), Glomerular Filtration Rate (**D**), and Creatinine (**E**).

Despite significant net diuresis, there was no adverse effect on renal function when comparing admission values to discharge values. Rather, there was a statistically significant improvement in creatinine, from 1.4 ± 0.5 mg/dL (123.8 ± 44.2 µmol/L) on admission to 1.2 ± 0.4 mg/dL (106.8 ± 35.4 µmol/L) on discharge (*p* < 0.05). Similarly, there was a significant improvement in GFR from 47.3 ± 12.1 mL/min/1.73 m^2^ on admission to 50.3 ± 11.1 mL/min/1.73 m^2^ on discharge, post diuresis (*p* < 0.05). There was no significant change in serum sodium on admission (137.3 ± 5.2 mmol/L) when compared to discharge (137 ± 3.9 mmol/L). In terms of hemodynamics, a decline in the mean arterial pressure from 85.6 ± 14.5 mmHg on admission to 78.5 ± 10.9 mmHg on discharge following large-volume diuresis (*p* < 0.05) was statistically significant. Jugular venous pressure examination significantly decreased from admission to discharge (15.8 ± 3.8 on admission to 8.7 ± 1.8 cm H_2_O on discharge) (Figure 2).

## 4. Discussion

This study demonstrated that in patients with severe precapillary PH presenting with acute decompensated RHF, significant intravenous diuresis had no significant adverse effect on renal function but rather led to the improvement of both creatinine and GFR. Additionally, there was no clinically significant adverse effect on hemodynamics when examining blood pressure before and after diuresis. These results mirror what has been anecdotally known by clinicians treating PH-related right heart failure, but this is the first objective evidence to support diuresis in the correct clinical context of precapillary PH patients.

Patients with RV dysfunction are often considered ‘preload dependent’, thus raising concern for hemodynamic and renal function compromise following extracellular volume removal [2,14,15]. This broadly applied concept likely stems from observations specific to the physiology of acute RV ischemia and infarction, where ischemia-induced RV dysfunction and loss of chamber compliance can lead to an altered RV pressure-volume relationship [16,17]. In these cases, with normal PVR, increased preload by way of modest volume loading is often necessary to maintain stroke volume and cardiac output until acute RV ischemia is alleviated. Similarly, acute preload reduction by way of diuresis or venodilation is not well tolerated hemodynamically in subjects with acute RV ischemia [16]. However, in cases of precapillary PH, the root cause of RV dysfunction is increased PVR and low PA compliance and, thus, increased RV afterload. Under these circumstances, volume loading is not effective in stroke volume recruitment but will lead to worsening right heart congestion, often leading to increased RV dilation, worsening TR, further leftward displacement of the interventricular septum, and overall hemodynamic deterioration [5,18,19,20]. 

In addition, unlike acute RV ischemia and infarction, the patients in our cohort presented with chronic PH-related right heart dysfunction and decompensated heart failure with clinical evidence of significant extracellular volume expansion. As such, in our cohort, intravenous diuresis was undertaken in the context of volume overload with the goal of restoring euvolemia and avoiding hypovolemia through careful serial assessment of right-sided filling pressures via JVP evaluation. Effective and appropriately administered diuresis in this context often has the opposite and, therefore, the beneficial effect of reduction in right heart filling pressure, RV dimension, and TR with ultimate hemodynamic improvement.

As management of RHF varies depending on its mechanism, we aimed to examine a group of patients with a robust precapillary PH phenotype, as evidenced by both echocardiographic data and hemodynamics from RHC. Precapillary PH has been defined as a mean PA pressure of >20 mmHg, along with a PVR of ≥3 WU, and a PCWP of ≤15 mmHg [9,10]. Our patient population fits this phenotype of precapillary PH not only by invasive hemodynamics on RHC but also with supportive findings on echocardiography. Further, we included patients with a PVR > 4 WU, which is higher than the normally defined cutoff. This was done to enrich the hemodynamic phenotype of the patient population with precapillary disease. While the cohort was mostly composed of Group I PAH patients (90.3%), there were six patients that had Group 3 PAH (9.7%). Despite a different WHO group categorization, Group 3 patients can have severe precapillary PH and right heart failure that is clinically indistinguishable from that of Group 1 patients in terms of hemodynamics and heart-failure-related presentations [21]. Of note, the average PVR of the Group 3 patients in this study was 11.8 WU, which was similar to the entire cohort. With a similar hemodynamic phenotype, Group 3 patients will require intravenous diuresis in the correct clinical setting, similarly to Group 1 patients. As such, the inclusion of Group 3 patients carries a similar relevance from a physiologic and clinical standpoint. Although chronic thromboembolic pulmonary hypertension (CTEPH), or Group 4 PH, also represents a cohort of precapillary PH patients, many of these patients were admitted for surgical management with pulmonary thromboendarterectomy. Further, there is a high use of intravenous contrast for the workup and management of CTEPH. As both points have the potential of significantly confounding the data, this group was excluded.

The severity of the precapillary PH phenotype is supported by the invasive hemodynamic data of the cohort, with a mean pulmonary artery pressure of 50 mmHg, PVR of nearly 12 WU, and a PCWP of only 10 mmHg [7]. There was a conspicuous absence of left heart disease in the cohort by echocardiography as well, given normal left ventricular systolic function, normal left atrial size, and an absence of left-sided valvular heart disease. In contrast, the majority of patients demonstrated severe RV dilation and RV systolic function. The presence of RVOT Doppler notching in a majority of subjects supports a high PVR and low PA compliance as the cause of PH in our cohort [22].

In this context, significant intravenous diuresis with an attendant volume and weight loss of 13 L and 7 kg, respectively, had no adverse effects on blood pressure or renal function. In contrast, there was a significant decrease in creatinine, a significant increase in GFR, and no significant change in serum sodium levels. Therefore, right heart decongestion with intravenous diuresis in this setting was not only safe but was directly beneficial to renal function. Improved renal function in this context is likely related to the direct relationship between right atrial pressure and renal venous pressure. Right heart decongestion and improved RA pressure translate to decreased renal venous pressure, subsequently leading to an improved renal arterial venous perfusion gradient. Diuresis was guided by daily assessment of right-sided filling pressures through JVP evaluation by experienced physicians within both the Heart Failure and PH departments and accomplished with relatively high doses of IV diuretics (Furosemide equivalents ranging from 40 mg/day to 3840 mg/day, Table 5). Despite the high doses of diuretics used to achieve euvolemia, there was still improvement in renal function. This was confirmed by examining a subset of patients who received particularly high doses of loop diuretics. There were 8 patients (12.9%) who received daily Furosemide equivalents of >1000 mg per day and accordingly diuresed 13.5 L on average, with an associated decline in JVP of >50%. This subset of patients had a decline in creatinine (1.8 to 1.4 mg/dL, 159.1 to 123.8 µmol/L) and an increase in GFR (37.8 to 46.1 mL/min), which was similar to the results of the entire cohort. As such, doses of diuretics did not have a detrimental effect on renal function as long as decongestion was achieved.

While the benefits of decongestion in left heart failure and cardiorenal syndrome have been studied extensively [23,24,25,26], the same is not true for right heart failure. Treatment guidelines for PH endorse the general use of diuretics in the setting of fluid retention; however, they do not provide specific recommendations nor cite evidence of their efficacy and safety in this specific cohort of patients with severe precapillary PH [7]. Further, there are no comments in the guidelines regarding the safety of diuresis in this cohort or the effects on renal function. Despite these general recommendations, it is not uncommon in clinical practice for these patients to present with hypotension and receive intravenous fluids in response. Contrary to this common clinical practice, the data from the current study supports the recommendations for the use of diuretics in a cohort of patients with severe PH and clinical right heart failure and, importantly, provides direct evidence of efficacy and safety in this clinical context.

Although we observed a modest drop in mean arterial pressure post-diuresis that proved statistically significant, this change was not clinically significant given the post-diuresis MAP remained well within a normal physiologic range, with no untoward clinical events and improved renal function. It is important to note that although MAP declined by 8% following diuresis, JVP (clinically estimated right atrial pressure) decreased by 46%. Recognizing that right atrial pressure is a close approximate to renal venous pressure, these findings lend toward a net increase in renal perfusion pressure, which likely in part explains the improved creatinine and GFR as in our cohort [26].

It is important to emphasize that this was a cohort of acutely decompensated patients, many of whom were presenting to our practice for the first time at the outset of initiation of their PH medical therapy. While a significant minority of patients were either on no PH medical therapy (22%) or PH monotherapy (19.3%) upon admission, this does not reflect their long-term PH therapy regimens. Changes were made to PH therapy regimens both during hospitalization and during short-term follow-up in the outpatient setting under the guidance of experienced PH experts. Notably, only two patients remained off all PH medical therapy at discharge, both limited by severe hypoxia in the context of Group 3 PH. While changes in the PH regimen were not the focus of our study, our observations of a lack of acquired azotemia and systemic hypotension in the context of often newly initiated PH medical therapy may strengthen our observations further. Specifically, it is notable that even in the context of severe precapillary PH presenting to us without a high incidence of dual or triple therapy, a relatively large volume IV diuresis was still not associated with clinically significant hypotension or azotemia.

## 5. Limitations

A potential limitation of the current study was the retrospective nature of the review, although the straightforward nature of our observations would not likely yield a different result if studied prospectively. Additionally, echocardiographic and hemodynamic data were not systemically repeated at the time of admission. However, echocardiographic and hemodynamic data were typically obtained in relatively close temporal proximity to admission. This, combined with the chronic nature of the condition, leads to the reported echocardiographic and hemodynamic data being representative of the patients’ physiologies at the time of admission. It is also important to note that the majority of patients with severe PH in this cohort either presented on PH medical therapy or were started on PH medical therapy during hospitalization. Although clinically appropriate and commensurate with the severe nature of their PH, our observations may have differed if the diuresis had been undertaken in the absence of PH therapy *OR* in the presence of an intensified PH regimen. Lastly, our observations should be interpreted with the understanding that the patients in our cohort were medically managed and diuresed at a major PH center under the care of experienced PH specialists.

## 6. Conclusions

In spite of anecdotal concern for preload dependence and hesitancy to diurese patients with PH and right heart dysfunction, this study demonstrated that significant intravenous diuresis in patients with severe precapillary PH and right heart dysfunction and clinical HF is not only safe but beneficial.

## Figures and Tables

**Table 1:** Demographics.

Demographics	Mean ± SD or N (%)
Age, y	64.7 ± 14.5
Sex	
Men	10 (16.1)
Women	52 (83.9)
Race	
White	38 (61.2)
Black	16 (25.8)
Hispanic	8 (12.9)
Other	10 (16.1)
Body Mass Index (kg/m^2^)	28.5 ± 8.1
NYHA Functional Class (noted closest to admission)	
I	2 (3.2)
II	5 (8.1)
III	33 (53.2)
IV	11 (17.7)
Other Medications	
ACE/ARB	8 (12.9)
MRA	17 (27.4)
CCB	4 (6.5)
Beta-blocker	21 (33.9)
Statin	26 (41.9)
Immunosuppressant	5 (8.1)
Co-morbidities	
Hypertension	31 (50)
Hyperlipidemia	9 (14.5)
Diabetes Mellitus	15 (24.2)
Chronic Kidney Disease	10 (16.1)
Chronic Obstructive Pulmonary Disease	16 (25.8)
Coronary Artery Disease	12 (19.4)
Atrial arrhythmias	12 (19.4)
Autoimmune Disease	23 (37.1)

Data are reported as either mean ± SD or number (percentage). Abbreviations: ACE—Angiotensin-converting enzyme, ARB—Angiotensin receptor blocker, CCB—Calcium channel blocker, IV—intravenous, MRA—mineralocorticoid receptor antagonist, NYHA—New York Heart Association.

**Table 2:** Pulmonary-hypertension-specific baseline characteristics.

Diagnosis-PH WHO Group	
PAH (Group 1)	56 (90.3)
PAH (Group 3)	6 (9.7)
PH Medical Regimens on Admission	
PDE5 inhibitor	44 (71)
Prostacyclin	30 (48.4)
Endothelin receptor antagonist	27 (43.5)
sGC Stimulator	8 (12.8)
Single	12 (19.3)
Dual	11 (17.7)
Triple	22 (35.4)
No PH Medical Therapy On Admission	17 (27.4)
Group 1	14 (22.5)
Group 3	3 (4.8)
No PH Medical Therapy On Discharge	2 (3.2)
Group 1	0
Group 3	2 (3.2)

Abbreviations: PAH—Pulmonary Arterial Hypertension, PDE5—Phosphodiesterase, sGC—Soluble guanylate cyclase.

**Table 3:** Echocardiographic parameters.

Echocardiographic Parameters				
Ejection Fraction (%)	60.9 ± 8.0			
PASP (mmHg)	79.2 ± 22.2			
TAPSE (cm)	1.4 ± 0.4			
Right Atrial Size	Normal 2 (3)		Dilated 60 (97)	
Left Atrial Size	Normal 50 (80.6)		Dilated 12 (19.4)	
Ventricular Septal flattening	None 4 (6.4)	Mild 6 (9.7)	Moderate 23 (37.1)	Severe 29 (46.8)
RVOT Systolic Notching	Present 42 (67.7)	Absent 12 (19.4)	Not evaluated 8 (12.9)	
RV Size (Dilation)	Normal 1 (1.6)	Mild 3 (4.8)	Moderate 17 (27.4)	Severe 41 (66.1)
RV Function (Degree of Dysfunction)	Normal 2 (3.2)	Mild 3 (4.8)	Moderate 24 (38.7)	Severe 33 (53.2)
Tricuspid Regurgitation (Degree)	None 3 (4.8)	Mild 10 (16.1)	Moderate 29 (46.8)	Severe 20 (32.3)
Pericardial Effusion (size)	None 33 (53.2)	Mild 19 (30.6)	Moderate 7 (11.3)	Severe 3 (4.8)

Abbreviations: PASP—Pulmonary artery systolic pressure, RV—Right Ventricle, RVOT—Right Ventricular Outflow Tract, TAPSE—Tricuspid annular plane systolic excursion.

**Table 5:** Admission characteristics.

Admission Characteristics			
Duration (days)		10.1 ± 6.4	
New PH Diagnosis on Admission		14 (22.5)	
30 day readmission rate		8 (12.9)	
Total Liters Diuresed (L)		13.3 ± 7.7	
Weight Change (kg)		−7.0 ± 6.7	
Mean highest daily diuretic dose (mg, Furosemide equivalents)		Median 240 (Min 20, Max 3840)	
**Diuretics Used**		**Number of Patients**	
Furosemide		25 (40)	
Bumetanide		41 (66)	
Torsemide		2 (3)	
Metolazone/Chlorothiazide		5 (8)	
**Pre and Post Diuresis (Entire Cohort)**	**Admission**	**Discharge**	***p* Value**
Systolic Blood Pressure (mmHg)	114 ± 20.6	106 ± 15.7	*p* < 0.05
Diastolic Blood Pressure (mmHg)	71.1 ± 13.5	64.9 ± 9.8	*p* < 0.05
Mean Arterial Blood Pressure (mmHg)	85.6 ± 14.5	78.5 ± 10.9	*p* < 0.05
Heart Rate (beats/min)	86.8 ± 16.1	79.7 ± 13.0	*p* < 0.05
Creatinine (mg/dL)	1.4 ± 0.5	1.2 ± 0.4	*p* < 0.05
GFR (mL/min/1.73 m^2^)	47.3 ± 12.1	50.3 ± 11.1	*p* < 0.05
Serum Sodium (mmol/L)	137.3 ± 5.2	137 ± 3.9	*p* < 0.3
Jugular Venous Pressure (cm H_2_O)	15.8 ± 3.8	8.7 ± 1.8	*p* < 0.05
**In Patients Receiving > 1000 mg** **Furosemide equivalents daily**	**Admission**	**Discharge**	***p* Value**
Mean Arterial Blood Pressure (mmHg)	87.3 ± 13.5	81.3 ± 12.4	*p* < 0.1
Creatinine (mg/dL)	1.8 ± 0.4	1.4 ± 0.5	*p* < 0.05
Glomerular Filtration Rate (mL/min/1.73 m^2^)	37.8 ±9.9	46.1 ± 14.1	*p* < 0.05

Data are reported as either mean ± SD or number (percentage). Furosemide equivalents: Oral: 1 mg Bumetanide = 20 mg Torsemide = 80 mg Furosemide. Intravenous: 1 mg Bumetanide = 20 mg Torsemide = 40 mg Furosemide [13].

## Data Availability

The data presented in this study is available on request from the corresponding author.
